# Safety management within the scope of teaching practical clinical skills: framing errors for cardiopulmonary resuscitation training – a multi-arm randomized controlled equivalence trial

**DOI:** 10.1080/07853890.2024.2408458

**Published:** 2024-10-07

**Authors:** Michelle Schmidt, Michael Tobias Schauwinhold, Leonie Anne Kathrin Loeffler, Martin Klasen, Sophie Isabelle Lambert, Saša Sopka, Lina Vogt

**Affiliations:** a AIXTRA – Competence Center for Training and Patient Safety, Medical Faculty, RWTH Aachen University, Aachen, Germany; b Department of Anaesthesiology, University Hospital RWTH Aachen, Medical Faculty, RWTH Aachen University, Aachen, Germany

**Keywords:** Error management, error culture, resuscitation, safety management, CPR training

## Abstract

**Introduction:**

Cardiopulmonary resuscitation (CPR) is among the most important skills in clinical practice. Errors can happen here, just like everywhere, and potentially have severe consequences. Two common error handling strategies known from practice are Error Management (EM) and Error Avoidance (EA). However, its effects on medical performance outcomes remain unclear. This study aimed to examine the role of error framing in basic life support (BLS) training for future healthcare professionals.

**Materials and Methods:**

In an equivalence trial (*N* = 430), first-year medical, dentistry, physiotherapy, and midwifery students underwent BLS training. In the three study arms, participants received either (1) instructions framing errors positively (EM), (2) instructions framing errors to be avoided (EA), or (3) no further instructions (Control). CPR performance was assessed using a resuscitation manikin measuring compression depth (CD) and compression rate (CR). The self-confidence ratings were assessed using a questionnaire. Equivalence margins for the outcome parameters and sample size calculations were based on previous standard BLS studies, using two-sided 95% confidence intervals to determine significance of equivalence.

**Results:**

The results regarding CD revealed equivalence with a trend toward superiority of EM over EA (proportional difference 23.3%-points; 95% CI 11.4%–34.2%) and EM over control (proportional difference 23.4%-points; 95% CI 11.5%–34.2%.) and significant equivalence of EA and control (proportional difference 0.1%-points; 95% CI −11.6%–11.7%). Significant equivalence was determined for all study arms with respect to CR and self-confidence.

**Conclusion:**

Our study revealed that EM was not detrimental to learners’ CPR performance. Given existing research on long-term beneficial effects of EM on patient safety, coupled with the proven equivalence of EM and EA concerning short-term performance, we argue that EM is a promising approach for future medical education purposes. Raising awareness of error framing and teaching error-handling strategies is expected to benefit ongoing safety management efforts in medical education and beyond.

## TWEETABLE SUMMARY

Study on error framing for CPR training: Promising implications for future healthcare education and safety management.

## Introduction

Medical error is a key aspect of patient safety [1]. There have been decades of effort concerning safety management and the role of errors in maximizing patient safety [1–5]. For example, Donchin et al. explored the nature and causes of human errors in the ICU by adopting a human factors engineering approach [4]. The findings suggest that strategies developed based on human factors engineering may reduce the incidence of errors in the ICU, thereby enhancing patient safety.

Safety management is defined as the absence of unwanted outcomes such as incidents or accidents, and includes regulation or control mechanisms [6]. Furthermore, it is related to the organization’s safety culture and includes organizational and behavioral elements of the system and its processes [7,8]. When examining medical error culture, medical students and trainees are hardly considered, although it can be assumed that they are an important target group for establishing an error culture that includes every individual within an organization [9,10]. 10.000 medical students complete their studies and enter the medical system in Germany annually, which underlines the relevance of including this target group [11]. Furthermore, a recent study showed that the error culture in German hospitals still has the potential for improvement [12]. Hence, organizational error culture might benefit greatly from addressing the role of errors during the early stages of medical education.

In the present study, we focused on two frequent paths for dealing with errors in professional life. First, Error Avoidance (EA), aims to avoid errors as much as possible [13]. From an EA perspective, errors are considered dispensable in terms of the learning process [14]. Since medical errors can have severe consequences and even be life-threatening, the overarching goal in medical practice is to ensure patient safety by reducing errors to a minimum [1,15,16]. Therefore, EA seems to be naturally promoted within the healthcare system [14]. The second path, Error Management (EM), fosters a positive approach to errors and attempts to stimulate learners by promoting errors as part of the learning process combined with active encouragement to make errors [14,17]. From an EM perspective, it is assumed that errors influence one’s own learning and enhance learning success [14,17]. In addition to negative effects, such as frustration, errors often provide useful information for learning how to deal with them in the future [18]. Therefore, EM focuses on reducing negative error consequences instead of the errors themselves [13,19]. EM is known for its beneficial effects on organizational learning and innovativeness, and its close connection with psychological safety and error culture by enabling team members to speak up and communicate errors effectively [3,20,21].

The empirically observed advantages of EM over EA can very well be embedded into a theoretical framework [22] which forms the scaffold for motivating the present study. The study’s framework is rooted in motivation theory; precisely, it is based on John W. Atkinson’s Choice Under Risk Model [23], an influential theory on the need for achievement. According to this theory, the need for achievement can be separated into two components, namely the motivation to achieve and the motivation to avoid failure. Besides dispositions of the acting person (traits), these two components are influenced by properties of the task, such as the difficulty and incentive value of success and failure. The relationship of these components to each other are important determinants of a person’s behavior in achievement situations – in particular, whether a person approaches a task optimistically or shies away from it fearfully. Concerning the framing (or labelling) of a task, as it was the intervention in our study, additional theoretical background comes from Goal Framing Theory [24]. Briefly, this theory highlights the importance of social contexts for individual motivation; in other words, how a person behaves in a certain situation depends on which ‘mind set’ is activated in the moment of the action. In our study, the ‘mind set’ was influenced by the EM/EA instruction. Based on the theoretical framework outlined above, we assumed that EM framing would increase the motivation to achieve, whereas the EA framework would increase the motivation to avoid failure.

Performance outcomes for the EA and EM approaches have been the topic of various studies, for instance, concerning software skills [5,17,18,25–27]. However, there is little research in the field of medical practice [14,28] and the findings are inconsistent [29]. Specifically, it is unclear whether EM instructions during training may lead to more performance errors – in other words, it still has to be shown that EM instructions are not detrimental to the learning success. Demonstrating such equivalence could also convince those practitioners who are hesitant to implement EM because of concerns about learners acquiring incorrect knowledge and the risk of internalizing erroneous patient handling [10,30]. This can be well investigated in learning with medical simulation; here, error-handling strategies can be practiced without restrictions because patient safety is not at risk [31].

To close the aforementioned research gap on EM and EA in the medical field, we conducted a study on the training of medical students in emergency skills. Sudden cardiac arrest is one of the most common causes of death worldwide [32,33]. In this event, basic life support (BLS) is the first immediate action to take. Cardiopulmonary resuscitation (CPR) skills represent core competencies in clinical practice and are essential for medical professionals to learn. Since the quality of CPR performance has a direct impact on saving lives, it may be one of the most important clinical practical skills to ensure patient safety [34]. However, a high rate of error in CPR application influences patient survival rates [35–37]. In addition to general ignorance of CPR actions, typical performance errors include too slow or too fast compressions and inadequate compression depth [38]. There has been much research tackling the challenge of teaching qualitative CPR, looking closer to the impact of different feedback and training concepts [39–42]. However, there is a research gap regarding the impact of different error-framing strategies on the acquisition of CPR skills.

As outlined before, EM has several advantages over EA in terms of long-term effects on psychological safety and error culture; however, its effect on short-term performance remains unclear. In the present study, we investigated the CPR performance of undergraduate medical students after training with either EM or EA instructions. Specifically, we sought to determine whether EM instructions lead to performance comparable to that of EA instructions.

## Materials and methods

### Trial registration

This study was registered with the ID DRKS00029981 at https://www.drks.de.

### Ethics

Ethical approval (Ethics Review Board document EK-22-290) was granted according to the ethical principles of the Declaration of Helsinki [43] of the World Medical Association on September 8^th^, 2022, by the Institutional Ethics Review Board of the University Hospital, RWTH Aachen University.

### Study design

**Figure 1.**
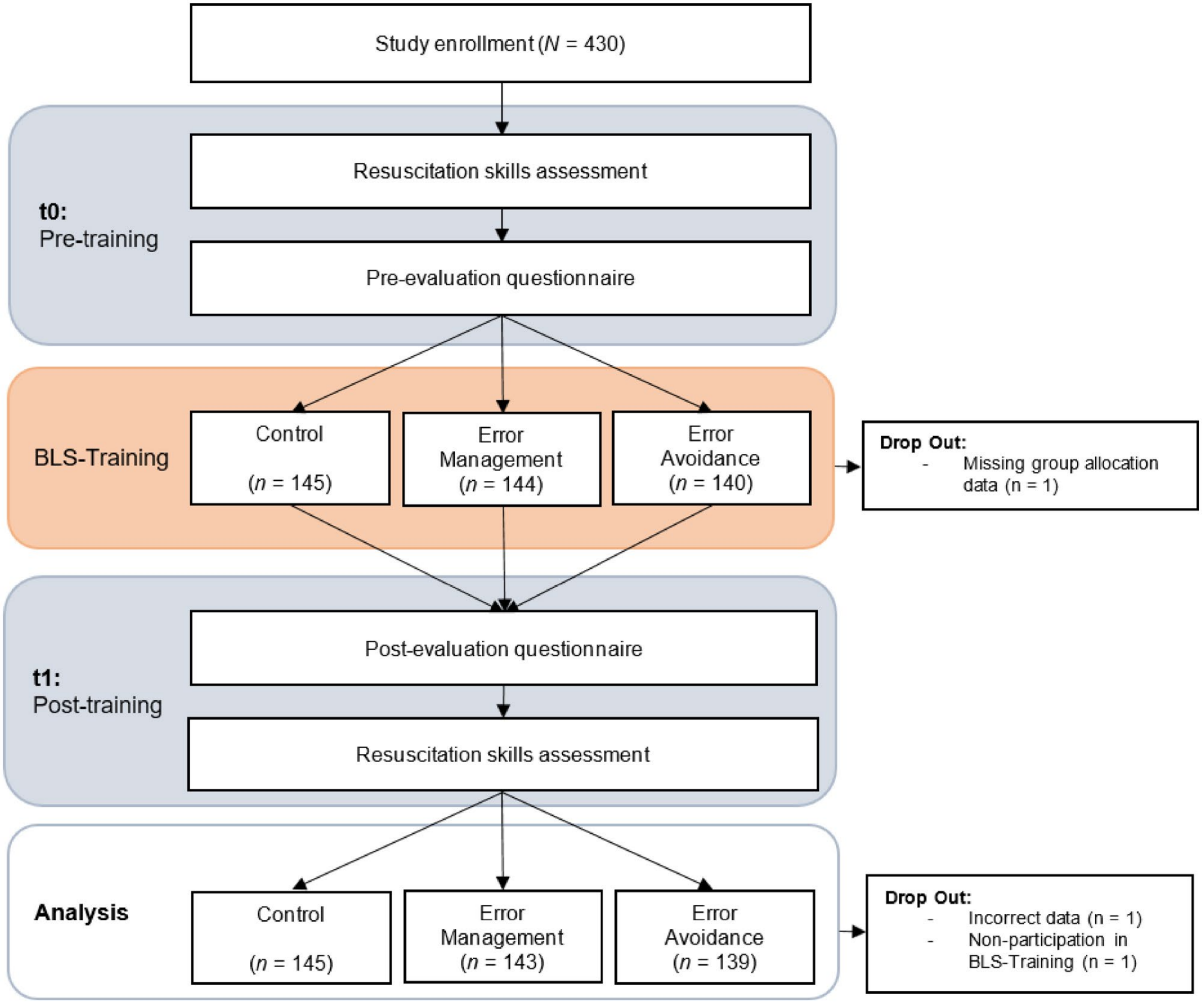
Flow chart of study design.

The multi-arm parallel-group randomized controlled trial consisted of three study conditions: (1) BLS training with EM instructions, (2) BLS training with EA instructions, and (3) BLS training with no further error instructions (control group) (see Figure 1) We adhered to the CONSORT guidelines for multi-arm parallel-group randomized trials as well as to the CONSORT guidelines for equivalence trials [44–46].

We chose an equivalence trial over a superiority trial for several reasons. EM offers benefits such as improving learning behaviour, organizational learning, and reducing negative error consequences. It also promotes psychological safety and open communication in teams [3,20,21]. These behaviours, in turn, are associated with increased error reporting and thus patient safety in nurses [47]. Despite these well-documented advantages in clinical practice, the equivalence of EM and EA in medical education remains uncertain. Concerns about learners internalizing erroneous knowledge and ethical considerations require investigation. Equivalence trials demonstrate interchangeability of interventions with similar effects on a specific variable [48]. Our study aimed to establish whether EM and EA result in comparable CPR performance outcomes. Equivalence designs are frequently used in the context of pragmatic (or practical) clinical trials [49]. More precisely, they are valuable tools when one of the interventions (in our case EM) has a clear advantage over the other, and one is interested in finding out whether it performs equally well with respect to another important outcome (in our case performance during learning). By exploring these outcomes, we aim to foster an academic discussion on implementing EM effectively in medical education and its broader impacts on error culture and, ultimately, patient safety.

### Participants

Participants were first-year undergraduate students in medicine, dentistry, physiotherapy, and midwifery. Data were collected during a mandatory introductory course on emergency medicine between October 12^th^ and October 27^th^, 2022. Written informed consent was obtained from all the participants. No exclusion criteria were defined to ensure a high ecological validity.

### Sample size planning

Sample size planning for equivalence testing was performed in accordance with Blackwelder [50] using a sealed envelope power calculator [51]. An α significance level of .05, a power (1-β) of 80%, and an estimated success of 46%, based on previous data sets from our institution, resulted in a required sample size of *N* = 230 (115 per group) for each comparison of two groups. These requirements were met for all the comparisons.

### Randomization

Groups of 12 participants were cluster-randomized to the study conditions (i.e. EA, EM, and control) using the Research Randomizer (https://www.randomizer.org/). Each study was conducted in a training room. The participants were allocated to the matching rooms according to randomization. There was no exchange among the participants concerning group allocation.

### Intervention

All study participants participated in a BLS training that was taught interprofessionally and according to the Peyton 4-step approach [52]. In the 1^st^ step, the correct BLS was demonstrated by a tutor at a normal pace without any comments (*Demonstration*). In the 2^nd^ step (*Deconstruction*), the tutor repeated the procedure and described every important aspect of the sub-steps of BLS. In the 3^rd^ step (*Comprehension*), the participants were asked to guide the tutor and provide instructions for the correct execution of BLS. The tutor would only take action if the participants provided instructions. In the 4^th^ and last step (*Performance*), the participants performed BLS and practiced their CPR skills on *ResusciAnne^TM^* while receiving feedback from a trained tutor. The manikin is a standard model for resuscitation training. Consisting of a torso and head with arms and legs, it offers trainees realistic resistance when practicing chest compressions. In addition, an audible click indicated the correct pressure depth. During the training, the tutors additionally provided a feedback device for the learners, which displayed the current compression rate with an indication of the correct frequency range, compression depth, and complete relief. Steps 1–3 were taught in a plenum of 36 participants in a large lecture hall, while for the last step, three groups (i.e. EA, EM, and control) with each 12 participants were formed. These groups went into individual training rooms according to randomization.

Before starting the 4^th^ step, the intervention groups (i.e. EM and EA) received additional instructions on how to handle errors. These instructions were presented in a specifically developed video for standardization purposes. In addition, standardized poster instructions highlighted key points from the videos and were visibly installed in the training rooms (see Supplementary Material). The instructions were based on the existing literature and adapted to the BLS training setting [5,18,25–27,53]. Video and poster instructions for the EM group framed errors as positive and necessary for effective learning (e.g. ‘*It can be expected that you will make errors while learning the resuscitation algorithm. Errors are an important part of the learning process.*’). The instructions for the EA group framed errors as to be avoided during the learning process (e.g. ‘*During the training you should try to avoid errors. Try to think in advance how you can avoid errors.*’). The third group (i.e. the control group) received no further instruction after the 3^rd^ step and was allowed to start with the 4^th^ step right after entering the training room.

### Primary outcome parameter: CPR skill assessment

CPR skills, a basic element of BLS training, were defined as the primary outcome parameter and assessed immediately before (t0) and one week after the training (t1) using a simulation manikin (ResusciAnneTM) by Laerdal, Stavanger, Norway. During the skill assessment, there was no use of an additional feedback device compared to the training time and no audible click indicated the correct compression depth. Participants received standardized instructions asking them to apply what they already knew about resuscitation (t0) or what they had learned (t1) before participating in the skills assessment. The tutors received standardized instructions on how to behave while monitoring the skills assessment. These included e.g. the request to not give feedback or any further information during skills assessment other than the initial instruction which reads as follows ‘*Please imagine that you see an unconscious person lying on the ground and come to help. Pretend that I am not there.*’. This ensured that every participant received the same information before and during the skill assessment. The scenario was terminated by the tutor two minutes after the first chest compression to ensure comparable compression times for all participants. CPR skills assessment included compression depth (CD) and compression rate (CR), recorded by the manikin’s Laerdal PC Skill Reporting System Software (Version 2.4.1, Laerdal, Stavanger, Norway). According to the American Heart Association (AHA) guidelines, correct CD was defined as an average CD between 50 and 59 mm, and correct CR was defined as an average CR of 100–120 compressions per minute [54].

### Secondary outcome parameter: subjective self-assessment

Participants were asked to fill out an online questionnaire before and after the BLS training, including demographic data as well as questions on the subjective confidence to perform CPR and deal with an emergency situation. Responses were given on a Likert scale ranging from 1 (totally disagree) to 6 (totally agree).

### Statistical analysis

Data were analyzed using IBM SPSS Statistics Version 28 (IBM Corp., Armonk, NY, USA). The application of equivalence analysis was recently discussed in the literature as a promising and appropriate tool to compare different teaching methods [49]. Therefore, equivalence was assessed by comparing the percentage of successful CD and CR performances after training in all three study arms. To determine equivalence, we used two-sided 95% confidence intervals (CI) according to the recommendations of the CONSORT statement [45]. Significance of results was given for 95% CIs of empirical percentage differences, excluding the equivalence margin values. The CIs for the differences between percentages were calculated using the Wilson score interval method for independent proportions [55]. Similarly, the 95% CI of the difference between the Likert scale confidence ratings in both study arms was used.

### Definition of equivalence margins

Previous research findings at our training center were used to define equivalence margins [56–58]. Rates of successful CPR after BLS training with Peyton’s 4-step approach were quantified for various samples of BLS-naive subjects, resulting in a covered range of 19 percentage points for CD (45%–64%) and CR (33%–52%). As this outcome variation was present with the standard approach, any outcome of another training method within these ranges was considered equivalent. Thus, for the comparison of EM, EA, and control, Δ = 19% and –Δ = −19% were defined as equivalence margins for both CD and CR. Since there was insufficient data available for confidence ratings, a difference of −0.5 points (approximately 8%) on the 6-point Likert scale was defined as the equivalence margin.

## Results

### Sample characteristics

In total, data were collected from *N* = 430 participants. Due to missing data on group allocation, incorrect data, or non-participation in the BLS training, three participants were excluded from further analysis. This led to a final study sample of *n* = 427 (70.7% female, 28.8% male, 0.5% diverse; age 20.7 ± 3.5 years). Partly completed datasets were included in the analysis. A chi-squared (χ^2^) test of independence was conducted to check if randomization was successful. The results indicated no significant differences in frequencies between the study groups in terms of demographic variables (see Table 1).

**Table 1.** Demographics of the study sample and randomization check.

	Control	Error management	Error avoidance	*X* ^2^	*p*
Sex (%)				1.93	.75
Female	67.6	72.3	72.4		
Male	31.7	27.0	27.6		
Diverse	0.7	0.7	–		
Age (M, SD)	20.7 ± 3.9	20.7 ± 3.7	20.8 ± 3.0	27.09	.94
Study program(%)				1.44	.96
Medicine	71.1	67.9	67.9		
Dentistry	14.8	16.1	15.7		
Physiotherapy	7.0	5.8	6.0		
Midwifery	7.0	10.2	10.4		
No previous medical qualification (%)	66.2	65.0	58.3	9.37	.90
Participation in an emergency course^a^ (%)	16.3	17.5	15.9	4.65	.91

*Notes. M* = Mean; *SD* = Standard deviation; ^a^ = participation in an emergency course within the previous year.

### Descriptive data

The pre- and post-training performance data and self-reported confidence ratings for all three study arms are shown in Table 2.

**Table 2.** Descriptive performance, target achievement and subjective confidence measures before (t0) and after (t1) the BLS training.

	Control			Error Management			Error Avoidance		
t0	*Md*	*IQR*	*NOR*	*Md*	*IQR*	*NOR*	*Md*	*IQR*	*NOR*
Ø CD (mm)	46.5	16	48	52.0	16	38	45.0	14	45
Ø CR (1/min)	102.0	23	83	103.0	25	105	107.0	23	119
Confidence for CPR performance	4.0	3	6	4.0	3	6	4.0	2	6
Confidence for emergency situation	3.0	3	6	3.0	3	6	3.0	3	6
	Achieved	*n*		Achieved	*n*		Achieved	*n*	
Correct CD (Total/%)	49 (34.5)	142		65 (46.4)	140		37 (27.2)	136	
Correct CR (Total/%)	62 (43.7)	142		59 (42.1)	140		67 (49.3)	136	
	Control			Error Management			Error Avoidance		
t1	*Md*	IQR	*NOR*	*Md*	IQR	*NOR*	*Md*	IQR	NOR
Ø CD (mm)	49.5	15	34	54.0	10	26	49.0	14	33
Ø CR (1/min)	100.0	21	66	99.0	17	81	101.0	16	94
Confidence for CPR performance	6.0	1	5	6.0	1	4	6.0	1	5
Confidence for emergency situation	5.0	1	6	5.0	1	5	5.0	1	6
	Achieved	*n*		Achieved	*n*		Achieved	*n*	
Correct CD (Total/%)	55 (40.4)	136		88 (63.8)	138		53 (40.5)	131	
Correct CR (Total/%)	64 (47.1)	136		59 (42.8)	138		60 (45.8)	131	

*Notes*. t0, pre-training assessment; t1, post-training assessment; CD = Compression depth; CR = Compression rate; CPR = Cardiopulmonary resuscitation; *M* = Mean; *SD* = Standard deviation; IQR = Quartile range; NOR = Non-outlier range.

### Equivalence analysis

**Figure 2.**
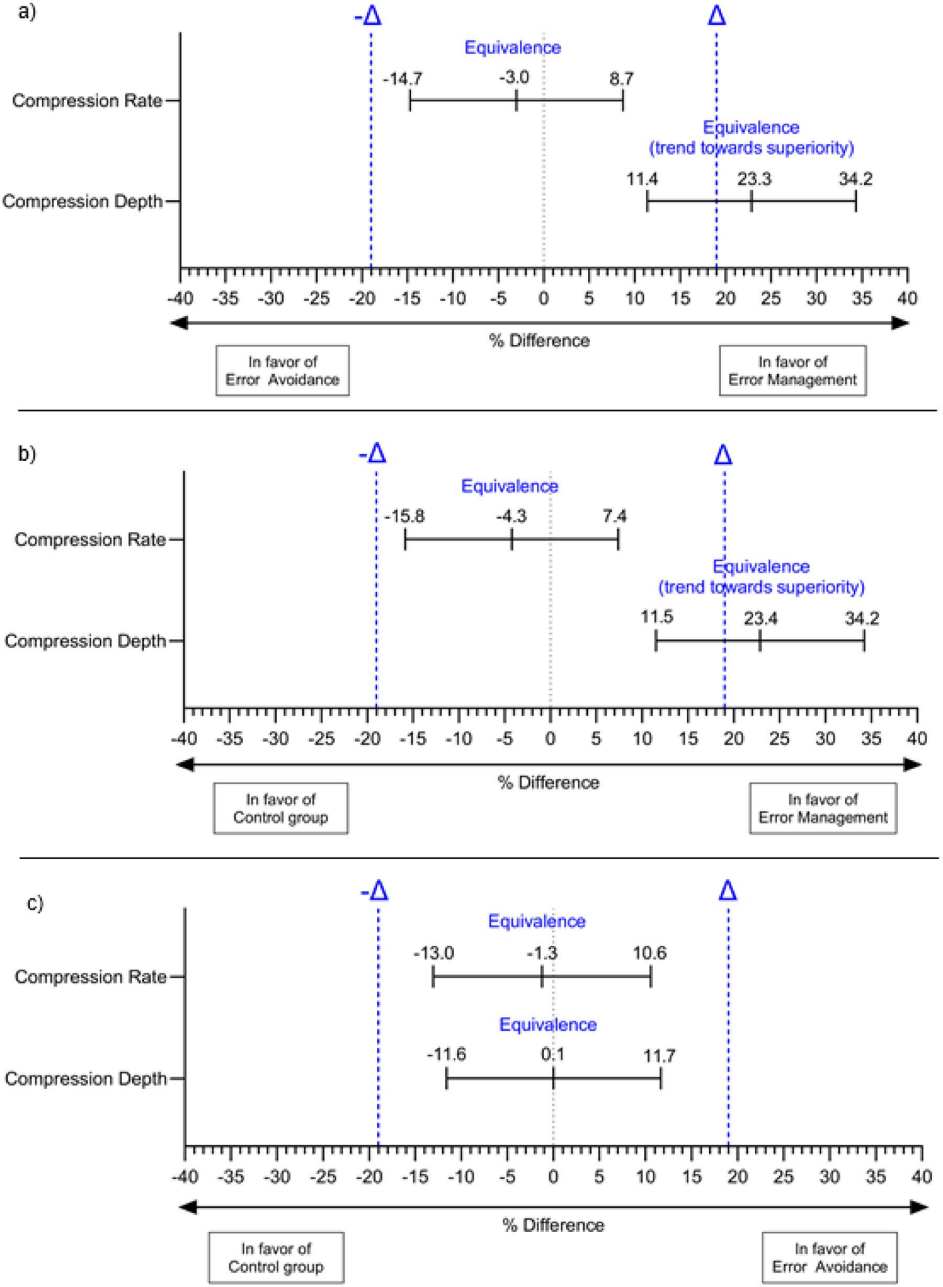
Equivalence analysis for the primary outcome parameter regarding the different error instructions (i.e. EM, EA, control). Notes. (a) displays the equivalence analysis for EA compared to EM; (b) shows the equivalence analysis for the control group compared to EM; (c) depicts the equivalence analysis for the control group compared to EA.

Results of equivalence analyses are reported in Figure 2 which displays the respective proportional differences between the EM, EA, and control groups at a 95% CI. The blue lines indicate the equivalence margins (-Δ and Δ).

### Compression depth

After the BLS training (t1), 40.4% in the control group, 63.8% in the EM group, and 40.5% in the EA group achieved the correct CD. The comparison of EA and EM results in a proportional difference of 23.3 percentage points (pp). The 95% CI for the proportional difference was 11.4% to 34.2%. The results indicate equivalence, with a trend towards the superiority of EM over EA. The comparison of the EM and the control groups resulted in a proportional difference of 23.4pp with 95% CI from 11.5% to 34.2%. The results indicated equivalence with a trend towards the superiority of EM over the control group. The comparison of the EA and control groups resulted in a proportional difference of 0.1pp. The 95% CI for the proportional difference was −11.6% to 11.7%. The results indicated significant equivalence between the EA and control groups.

### Compression rate

After the BLS training (t1), 47.1% in the control group, 42.8% in the EM group and 45.8% in the EA groups, achieved correct CR. The comparison of the EA and EM groups resulted in a proportional difference of 3.0 percentage points (pp). The 95% CI for the proportional difference was −14.7% to 8.7%. The results indicated significant equivalence between the EM and EA groups. The comparison of the EM and control groups resulted in a proportional difference of 4.3pp with 95% CI from −15.8% to 7.4%. The results indicated significant equivalence between the EM and control groups. Comparison of the EA and control groups resulted in a proportional difference of 1.3pp. The 95% CI for the proportional difference was −13.0% to 10.6%. The results indicated significant equivalence between the EA and control groups.

### Subjective self-assessment

**Figure 3.**
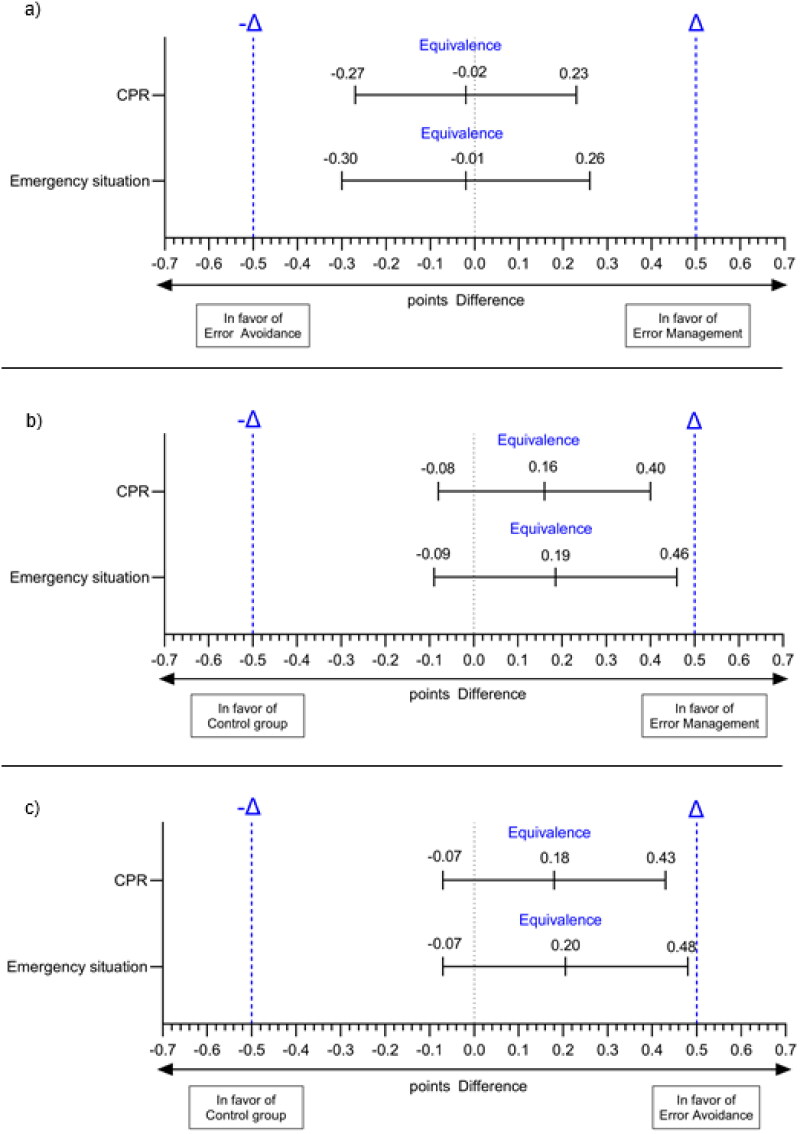
Equivalence analysis for the secondary outcome parameter regarding the different error instructions (i.e. EM, EA, control). *Notes*. (a) displays the equivalence analysis for EA compared to EM; (b) shows the equivalence analysis for the control group compared to EM; (c) depicts the equivalence analysis for the control group compared to EA.

Mean differences (t1) between the EM and EA group were 0.02 points (95% CI: −0.27 to 0.23) regarding confidence in one’s CPR performance and 0.01 points (95% CI: −0.30 to 0.26) in terms of confidence in an emergency situation. The results indicated significant equivalence between the EM and EA groups for both items. Mean differences (t1) between the EM and control group were 0.16 points (95% CI: −0.08 to 0.40) regarding confidence in one’s CPR performance and 0.19 points (95% CI: −0.09 to 0.46) in terms of confidence in an emergency situation. The results indicated significant equivalence between the EM and control groups for both items. Mean differences (t1) between the EA and control group were 0.18 points (95% CI: −0.07 to 0.43) regarding confidence in one’s CPR performance and 0.20 points (95% CI: −0.07 to 0.48) in terms of confidence in an emergency situation. The results indicated significant equivalence between the EA and control groups for both items. The results are presented in Table 2 and Figure 3.

## Discussion

The impact of different error-handling strategies, such as EM and EA, on medical performance outcomes in learning situations has rarely been studied. Therefore, we examined the equivalence of EM and EA instructions regarding CPR performance in the context of medical education. Our study indicates equivalence with a trend towards superiority of EM over EA and control with regard to CD. This refutes the assumption that promoting errors as an essential part of the learning process can result in learners making more errors. Instead, our results show that in the context of medical education, performance is comparable when explicitly encouraged to try out and make errors compared to avoiding errors or receiving no specific error-related instructions. One reason for this effect might be that EM encourages learners to experiment, which helps them to gain a better understanding of the correct CD [21]. Another reason might be that EM contributes to a safe learning environment and creates psychological safety. Psychological safety is described as a condition in which learners do not have to fear judgement in the eyes of others or negative consequences depending on their performance [59]. By creating psychological safety, participants might have felt safer to perform CPR, which led to fewer errors and, consequently, better performance outcomes. However, the lack of psychological safety in the EA and control groups may have impaired performance [60]. However, this effect could also be partly attributed to prior performance differences between the study arms, which might have biased the results.

Furthermore, our results reveal a significant equivalence of the EA and control groups with regard to CD. This is an interesting result, as it shows that no instruction or instructions to strictly avoid errors produce comparable results. Avoiding certain errors at all costs is expected to have an impact on patient safety [1,61]. However, according to our results, comparable effects would occur with no further instructions when examined in the context of medical simulation, where patients’ lives are not at risk. This calls into question whether EA instructions can be useful and beneficial for future medical education purposes. However, it questions whether standard practice in medical care, which generally highlights the importance of avoiding errors to maximize patient safety [1,14–16], is as effective as we think. Future research should consider examining a combination of EM and EA to determine the positive effects of both instructions.

In terms of CR, our results show significant equivalence for all study arms (i.e. EA, EM, control), meaning that being either instructed to avoid errors (EA), being encouraged to try out and make errors (EM), or receiving no instruction (control group) led to comparable performance. This effect might be due to being more familiar with maintaining a certain rate than achieving the correct CD. In terms of CR, it may be easier to orient oneself to a certain rhythm that can either be too fast or too slow, whereas the correct CD is influenced by multiple factors, such as the correct hand position [62] or the body weight of the person applying CPR [63]. The study by Hafner et al. [64] provides evidence that incorporating a song as a metronome is a practical and easily implementable method for training individuals to achieve the appropriate CR during CPR. While achieving the correct CD is influenced by various factors, including proper hand position and the body weight of the individual administering CPR [62], we argue that maintaining the correct CD may pose greater challenges compared to maintaining the correct CR.

Furthermore, the results on self-confidence to be able to provide CPR and deal with a non-responsive person in an emergency situation revealed significant equivalence for all study arms (i.e. EA, EM, control). These results suggest that the different error instructions and no error instruction led to comparable self-confidence in providing CPR or dealing with an emergency situation. While we assumed that EA instructions could have negatively affected psychological safety, which might have impaired performance outcomes for CD [60], this does not seem to be the case when looking at self-confidence ratings. However, further research is needed to examine the effects of error handling and psychological safety more closely.

In summary, safety management and the role of errors are important aspects that should be considered in medical education. This study serves as a first step toward being explicit about the equivalence of different error-framing instructions for practical clinical skills obtained in BLS training and what might be an effective way to form a beneficial error culture during the early steps of medical education.

### Limitations

To the best of our knowledge, this is the first study to investigate the effects of framing errors in a three-armed study design, focusing on CPR skills. However, the results of this study should be interpreted in light of some limitations. Because the study focused on the field of medical education, the results cannot be generalized to medical practice or other clinical skills. Future research needs to examine whether comparable effects would occur outside of a simulation setting as well as for other clinical skills. Our results do not allow any conclusions to be drawn regarding the long-term effects of certain error-framing instructions. Moreover, the error instructions used in our study provide a resource-efficient way to introduce the role of errors in medical education. The study intervention was rather short, and it was not recorded whether or how long participants paid attention to the instructions. Therefore, the question arises as to what a sufficient number and length of instruction is required to induce a sustainable error culture. Future studies should elaborate on this topic and identify the threshold at which different error instructions are equivalent. Furthermore, the error instructions used in this study were based on previous studies that developed and implemented them. However, these error instructions have not been validated before which should be taken into account when interpreting the study results. A further limitation of the study represents the validation of the items concerning demography and subjective feeling of safety used in the questionnaire. For these items, solely face validity can be reported which should be taken into account when interpreting the results. The present study focused on how different error instructions influenced the CPR performance. An interesting aspect for future studies could be to examine how feedback based on the respective error instructions influences the CPR performance.

## Conclusion

Safety management and error culture in medical care are important aspects of addressing medical errors, relevant to both qualified healthcare professionals and medical education. Our study shows that different ways of error framing (i.e. EM and EA) lead to comparable CPR performance. EM demonstrates a trend towards superior performance in CD while remaining statistically equivalent to EA in CR and self-reported confidence. Given existing research on long-term beneficial effects of EM on patient safety, coupled with the proven equivalence of EM and EA concerning short-term performance, we argue that EM is a promising approach for future medical education purposes. Our results are expected to impact how the inducement of an error culture is perceived in medical practice, as well as in medical education and beyond. Further methods to integrate the role of errors must be examined.

## Supplementary Material

Supplementary Material_Error Framing Instructions.docx

## Data Availability

Study material and datasets are available from the corresponding author upon reasonable request.
