# Supplementary material for: Safety management within the scope of teaching practical clinical skills: framing errors for cardiopulmonary resuscitation training – a multi-arm randomized controlled equivalence trial
Source: Ann Med. 2024 Oct 7;56(1):2408458. doi: 10.1080/07853890.2024.2408458 (PMC11459762; doi:10.1080/07853890.2024.2408458)
Supplement: Supplementary Material_Error Framing Instructions.docx [file IANN_A_2408458_SM0015.docx]

**Supplementary Table.** Error Framing Instructions

| **Poster Instructions** | | |
| --- | --- | --- |
|  | **Original German Version** | **Translated English Version** |
| **Error Management** | Ich habe einen Fehler gemacht. Prima!  Fehler sind ein natürlicher Teil des Lernprozesses. Sie zeigen auf, was ich noch lernen kann!  Ich versuche mehr zu lernen, indem ich Fehler zulasse! | I made an error. Great!  Errors are a natural part of the learning process. They show what I can still learn!  I try to learn more by allowing errors to happen! |
| **Error Avoidance** | Ich habe einen Fehler gemacht. Nächstes Mal vermeide ich das!  Fehler sollten während des Lernprozesses vermieden werden!  Ich versuche mehr zu lernen, indem ich Fehler vermeide! | I made an error. Next time I will avoid it!  Errors should be avoided during the learning process!  I try to learn more by avoiding errors! |
| **Video Instructions** | | |
|  | **Original German Version** | **Translated English Version** |
| **Error Management** | Liebe Teilnehmerin, lieber Teilnehmer,  in Kürze beginnt dein Basic Life Support Training. Hierzu noch ein paar wichtige Informationen vorab.  Es ist zu erwarten, dass du beim Erlernen des Reanimationsalgorithmus Fehler machst. Fehler sind ein wichtiger Bestandteil jeder Lernerfahrung. Das Auftreten von Fehlern kann sehr nützlich sein, denn aus Fehlern kann man viel lernen. Sie lenken die Aufmerksamkeit bewusst auf Dinge, denen man sich zuvor nicht bewusst war. Wenn du einen Fehler gemacht hast, ist es wichtig, darüber nachzudenken, wie du ihn korrigieren kannst, was du daraus lernen kannst und wie das Gelernte dir in Zukunft in ähnlichen Situationen helfen kann.  Viel Erfolg bei deinem Training. | Dear Participant,  your Basic Life Support Training will begin shortly. Here is some important information in advance.  It is expected that you will make errors while learning the resuscitation algorithm. Errors are an important part of any learning experience. Making errors can be very useful because you can learn a lot from errors. They consciously draw your attention to things you were not aware of before. If you have made an error, it is important to think about how you can correct it, what you can learn from it, and how what you have learned can help you in similar situations in the future.  Good luck with your training. |
| **Error Avoidance** | Liebe Teilnehmerin, lieber Teilnehmer,  in Kürze beginnt dein Basic Life Support Training. Hierzu noch ein paar wichtige Informationen vorab.  Während des Trainings solltest du versuchen, Fehler zu vermeiden. Du solltest immer versuchen, die Reanimation korrekt zu erlernen und keine Fehler zu machen, damit das Training möglichst effektiv ist. Deshalb solltest du versuchen, im Voraus zu überlegen, wie du Fehler vermeiden kannst. Überlege, was du tun kannst, um Situationen, die zu Fehlern führen, erfolgreich zu erkennen.  Viel Erfolg bei deinem Training. | Dear Participant,  your Basic Life Support Training will begin shortly. Here are some important information in advance.  During the training you should try to avoid errors. You should always try to learn resuscitation correctly and not make errors so that the training is as effective as possible. Therefore, you should try to think in advance how to avoid errors. Think about what you can do to successfully recognize situations that lead to errors.  Good luck with your training. |
